# The practice of child and adolescent psychiatry: a survey of early-career psychiatrists in Japan

**DOI:** 10.1186/1753-2000-3-30

**Published:** 2009-09-28

**Authors:** Masaru Tateno, Naoki Uchida, Saya Kikuchi, Ryosaku Kawada, Seiju Kobayashi, Wakako Nakano, Ryuji Sasaki, Keisuke Shibata, Tomohiro Shirasaka, Muneyuki Suzuki, Kumi Uehara, Toshikazu Saito

**Affiliations:** 1 Department of Neuropsychiatry, Sapporo Medical University, School of Medicine, Sapporo, Japan; 2 Department of Psychiatry, Fukuoka University, School of Medicine, Fukuoka, Japan; 3 Department of Psychiatry, Tohoku University, Graduate school of Medicine, Sendai, Japan; 4 Department of Neuropsychiatry, Graduate School of Medicine, Kyoto University, Kyoto, Japan; 5 Department of Psychiatry, University of Occupational and Environmental Health, Kitakyushu, Japan; 6 Division of Neuropsychiatry, Sunagawa City Medical Center, Sunagawa, Japan; 7 Department of Psychiatry, Graduate School of Medical Science, Kyoto Prefectural University of Medicine, Kyoto, Japan; 8 Department of Neuropsychiatry, Kyushu University Hospital, Fukuoka, Japan; 9 Kanagawa Psychiatry Medical Center Serigaya Hospital, Yokohama, Japan

## Abstract

**Background:**

Child and adolescent psychiatry (CAP), a subspecialty of psychiatry in Japan, is facing a serious workforce shortage. To resolve this situation, the Japanese government has organized a task force and has been working to increase psychiatrists' clinical skills to improve care for children and adolescents with mental health problems. Using an online questionnaire system, the authors have conducted a survey to investigate the perceptions, experiences, and interests of early-career psychiatrists in CAP.

**Methods:**

The subjects of this study were 182 psychiatrists in Japan whose individual clinical experiences did not exceed 15 years. The authors of this study created an online questionnaire system and e-mailed the URL and login password to all subjects. Respondents anonymously answered the questions. Most questions required an answer indicating a level of agreement scored on a nine-point scale. Responding to the questionnaire was considered to constitute consent, and all respondents' privacy was carefully protected.

**Results:**

The mean age and clinical psychiatric experience of the subjects were found to be 33.1 ± 4.5 years and 5.43 ± 3.5 years, respectively. On a nine-point scale (with nine being the highest), experience and interest in CAP measured 3.05 ± 1.9 and 5.34 ± 2.5, respectively; further, these two factors showed significant correlation (r = 0.437, p < 0.0001). The mean score for the early-career psychiatrists' confidence in their ability to diagnose and appropriately treat was notably low, at 3.13 ± 1.9.

**Conclusion:**

Our results demonstrated that early-career psychiatrists self-evaluated their CAP clinical experience as insufficient, and these clinicians' CAP experiences and interests correlated significantly. Therefore, in order to improve child and adolescent medical care, we need to expose young psychiatrists to sufficient CAP cases and explore the factors that could attract them to this field.

## Background

In Japan, child and adolescent psychiatry (CAP) is not a separate specialty; instead, it is considered a psychiatric subspecialty [1]. There is no standardized residency program in the field, and each teaching hospital determines its own curriculum. As a result, CAP training content and clinical experience varies greatly among institutions [2].

Instead of a uniform residency program in CAP, the Japanese Society for Child and Adolescent Psychiatry (JSCAP; ), established in November 1960, initiated its own certification system in April 1992 which requires: 1) over five years of clinical experience in medicine including over two years in general psychiatry and over three years in CAP, 2) being a member of this society for over five years, 3) passing an examination, 4) a list of 30 CAP cases seen in the preceding three years, and 5) three case reports (at least one must be a case involving a developmental disorder). In Japan, the clinician certified by the JSCAP is regarded as a specialist in CAP. However, to date, only about 150 clinicians have been certified as specialists by JSCAP. Since the latest data provided by the Japanese Ministry of Health, Labor and Welfare (JMHLW) reports that the total number of psychiatrists in Japan was 12,474 in 2006 (on-line database of JMHLW; ), the number of CAP specialists is remarkably low.

The increasing rate of mental and developmental problems among the younger generation has attracted the attention of both laypeople and medical professionals. Indeed, the suicide rate among young adolescents [3,4], school refusal and school absenteeism [5,6], and the phenomenon of social withdrawal [7] are all continuing to gradually increase. Tsuchiya et al. reported that the age-adjusted suicide rate in 2000 was 1.1 and 6.4 per the corresponding 100,000 population for children aged 10-14 and 15-19, respectively [1]. Lately, the mass media has often highlighted the suicides of students who have been bullied at school or on the Internet. These cases have been known as *net suicides *[4], and CAP professionals have worked to intervene and attempt prevention. Despite increasing social demands and a need to increase the number of clinicians who could address children's mental health problems, Japan continues to face a serious shortfall in its CAP workforce.

Previous studies have demonstrated that early exposure to certain psychiatric subspecialties during residency training can positively impact career choices [8,9]. However, the reality is that most young psychiatrists who start out interested in CAP lose motivation and change their career path to another speciality or subspecialty without having had sufficient CAP experience [10].

**Figure 1:**
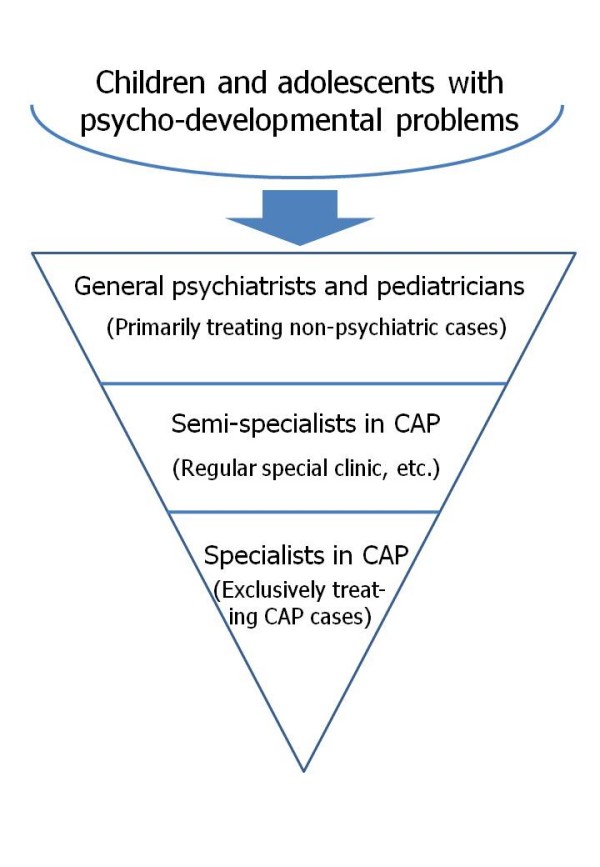
**A three-tier CAP medical workforce structure**. The task force established by the Japanese Ministry of Health, Labour, and Welfare classified those psychiatrists/paediatricians who work with child and adolescent mental health problems into three groups; the task force has provided objectives and training opportunities for each group to work toward resolving the serious shortfall in this field's workforce.

In March 2005, in response to these social demands and the corresponding urgent need to add to the number of clinicians who can treat children with mental health problems, JMHLW established a task force to educate clinicians about CAP. The task force proposed a three-tier CAP medical workforce structure as shown in Figure 1. General psychiatrists and paediatricians are at the pinnacle of the inverted triangle, and they play an important role as the gatekeepers to appropriate medical intervention. Psychiatrists and paediatricians who periodically treat the psychiatric-developmental problems of children and adolescents could be assigned to the middle tier as CAP semi-specialists. In fact, many medical professionals in this subgroup have regular CAP clinics and provide a certain level of inpatient care. The third tier involves those psychiatrists and paediatricians who almost exclusively treat child and adolescent patients. Most psychiatrists and paediatricians who fall within this group are practicing as specialists at hospitals with CAP wards. The JMHLW task force set learning objectives for each of these three categories of psychiatrists and paediatricians and has been providing various training opportunities such as case conferences, seminars, workshops, etc. in collaboration with academic societies in this field. The task force works to enhance the basic clinical skills of all clinicians and also encourages the psychiatrists and paediatricians at the pinnacle of the inverted triangle to move down to the middle tier in the inverted triangle model. Nevertheless, Japan continues to face a serious shortfall in its CAP workforce.

Career choice among early-career psychiatrists can be affected by a variety of factors [11-15]. Previous studies have demonstrated that intensive early exposure to a psychiatric subspecialty could affect the career decisions of psychiatric residents [8,9]. To assess the CAP experiences and interests among young psychiatrists in Japan, the authors conducted an online survey to explore the factors that could affect early-career psychiatrists' decisions about whether to select CAP as a subspecialty.

## Methods

### Subjects

The subjects of this study were early-career psychiatrists in Japan. In this context, the early-career psychiatrist is defined as a psychiatrist whose clinical experience does not exceeded 15 years. Study collaborators were recruited through the listserv application of the Japan Young Psychiatrists Organization (JYPO; ), and those collaborators in turn encouraged their colleagues to participate in the survey. The study authors created an online questionnaire and e-mailed the URL and login password for that questionnaire to all collaborators. The study collaborators then distributed the invitation e-mail to their colleagues. All subjects were requested to complete the questionnaire within the survey period, which was February 20 through April 20, 2009. The Fukuoka University Hospital's ethics committee approved the study protocol. The study's aim was clearly stated on the online survey system's main web page and answering the questionnaire was deemed to constitute consent. All respondents participated in this study without any incentive. Similarly, all authors and subjects involved in this study declared themselves free of any conflict of interest relating to the study.

### Questionnaire

The questionnaire consisted of twenty-two questions divided into six categories: (1) demographic information; (2) future subspecialty preference; (3) subspecialty experience; (4) attitude toward subspecialties; (5) attitude toward geriatric psychiatry; (6) attitude toward CAP; and (7) attitude toward alcohol and addiction.

The survey contained three types of responses: open responses, single and multiple-choice responses, and responses on a nine-point Likert scale. A Likert scale is one of the most commonly used methods for the measurement of attitudes in various surveys [16-18]. We asked the subjects to rate their answers by using a nine-point scale which was slightly modified from a format used in an expert consensus guideline series [19]. Briefly, the subjects were asked to respond to a statement about their level of CAP experience using a nine-point scale ranging from one, indicating complete insufficiency, to nine, indicating appropriate sufficiency, with five representing a neutral response that neither agreed nor disagreed with the statement. Similarly, each respondent was asked to self-evaluate, on a nine-point scale (again, with nine representing the highest agreement with any given proposition), his or her interest, expert knowledge, confidence in diagnosis and treatment, sense of satisfaction, potential primary practice interest, and optimism about the future of the CAP field. Furthermore, respondents were surveyed about their interest in pervasive developmental disorders (PDD), whether that interest in PDD was focused toward clinical work or was research-oriented, about any difficulties experienced in distinguishing between PDD and schizophrenia, about interest in child and adolescent cases with schizophrenia or mood disorders, about interest in working with children, and about their understanding of normal childhood development.

In this paper, we have confined our report to the results based on questions about CAP. Survey results regarding other subspecialties, such as geriatric psychiatry, will be reported elsewhere.

### Statistical Analysis

Study results were expressed as the mean ± SD. Statistical analysis was performed using SPSS 16.0J for Windows (SPSS Japan Inc., Tokyo, Japan). The statistical significance was set at a *p *value of less than 0.05.

## Results

### Subject demographics

A total of 200 psychiatrists answered this study's questionnaire. Because we used the previously described online questionnaire system for data collection, it was not possible to calculate a precise response rate. One of the factors which complicated the response rate calculations of the data collection through the Internet is the fact that some of mailing lists used in this study contained a number of invalid addresses. However, based on the estimated response rate reported from each site investigator, we estimate the total response rate at 85 percent.

Psychiatrists whose individual clinical experience exceeded 15 years were excluded from the statistical analysis (n = 18, 9.0 percent of all respondents) because this study's goal was to gain insight into early-career psychiatrists' perceptions. Thus, the number of subjects totalled 182 and their detailed demographics are summarized in Table 1. The mean age and clinical psychiatric experience were 33.1 ± 4.5 years and 5.43 ± 3.5 years, respectively.

**Table 1:** Demographics of early-career psychiatrists

	**Total**
Subjects	182
	
Gender	
Male (%)	131 (72.0)
Female (%)	51 (28.0)
	
Age	33.1 ± 4.5 y
<30 year old (%)	35 (19.2)
30-39 years old (%)	135 (74.2)
≥ 40 years old (%)	12 (6.6)
	
Work setting	
University hospital (%)	96 (52.7)
General hospital (%)	24 (13.2)
Private psychiatric hospital (%)	52 (28.6)
Private psychiatric clinic (%)	5 (2.7)
Other (%)	5 (2.7)
	
Clinical experience in psychiatry	5.43 ± 3.5 y
1st year	30 (16.5)
2nd year	21 (11.5)
3rd year	21 (11.5)
4 - 5th year	16 (8.8)
6 - 7th year	43 (23.6)
8 - 10th year	37 (20.3)
11th - year	14 (7.7)

The age and psychiatric clinical experience are indicated as the mean ± SD. Other results are shown both as a number and as a control percentage in parentheses.

### Self-evaluation of experience and interest in CAP

The results of self-evaluation of experience and interest in CAP were summarized in Table 2. On a nine-point Likert scale (with nine being the highest possible score), the mean experience and interest were 3.05 ± 1.9 and 5.34 ± 2.5, respectively. The term interest in this context included a sense of concern with this area, as well as a motivation to pursue it with higher priority and an interest in getting further training in this field. Statistical analysis revealed a significant correlation between experience and interest in CAP (r = 0.437, p < 0.0001, Spearman rank correlation). Two group comparisons demonstrated that the interest in CAP was higher among women (6.30 ± 2.5) than men (4.97 ± 2.4, p = 0.001, Student's t-test). The subjects expressed lower interest in pervasive developmental disorders (5.39 ± 2.4) than they did in child and adolescent mood disorders and schizophrenia (6.01 ± 2.2). Our results reported a remarkably low rate of self-confidence among early-career psychiatrists in their ability to diagnose and make appropriate interventions in CAP cases (3.13 ± 1.9). The subjects also rated their understanding of normal childhood development at 4.66 ± 1.9 on the nine-point scale, which suggests that they viewed an insufficient understanding of developmental psychology as their collective weak point.

**Table 2:** CAP self-evaluation (n = 182)

	**mean ± SD**
Interest in CAP	5.34 ± 2.5
Experience with CAP	3.05 ± 1.9
Knowledge of CAP	3.43 ± 1.9
	
Confidence in diagnosing/treating CAP cases	3.13 ± 1.9
Potential for CAP as career choice	4.15 ± 2.5
Estimated sense of satisfaction in a CAP career	5.45 ± 2.2
Bright future with CAP as a subspecialty	5.72 ± 2.0
	
Fondness of children	6.35 ± 2.3
Understanding of normal childhood development	4.66 ± 1.9
	
Interest in schizophrenia and mood disorders in CAP	6.01 ± 2.2
	
Interest in PDD	5.39 ± 2.4
Dominant clinical interest (percentage)	117 (64.3)
Dominant research interest (percentage)	45 (24.7)
Equal interest in clinical and research (percentage)	18 (9.9)
Difficulties in diagnosing PDD	6.60 ± 2.1

These results (scored on a nine-point scale with nine being the highest possible score) are expressed as the mean ± SD.

## Discussion

Our results revealed that an interest in CAP significantly correlated with experience in the field (r = 0.437, p < 0.0001, Spearman rank correlation). A distinct difference was found on a nine-point scale between self-evaluated CAP interest (5.34 ± 2.5) and self-evaluated CAP experience (3.05 ± 1.9). The considerably low scores on CAP knowledge (3.43 ± 1.9) and confidence about the ability to diagnose and treat appropriately (3.13 ± 1.9) could be explained by insufficient CAP experience in the early stages of the respondents' training. The score quantifying the probability of CAP as a subspecialty choice was 4.15 ± 2.5. These results suggest that limiting early-career psychiatrists' exposure to CAP could dissuade them from this subspecialty, even though the subjects thought that CAP, as a psychiatric subspecialty, could be somewhat satisfying and would have a relatively bright future.

Of the 182 subjects, 105 (57.7 percent) answered that they liked children, scoring seven or higher on this question, and the mean was 6.35 ± 2.3. It would seem reasonable to assume that those who like children would be likely to choose an occupation related to children. Interest in CAP significantly correlated with the indicated degree of liking children (r = 0.448, p < 0.0001, Spearman). However, the correlation between the extent to which a respondent self-evaluated being fond of children and the self-evaluated possibility of choosing CAP as a career was somewhat weaker (r = 0.370, p < 0.0001, Spearman).

In the clinical practice of CAP in Japan, subjects with developmental disorders account for a considerably high percentage among those seeking health care [20,21]. Recent epidemiological studies on PDD demonstrate a steep increase of its prevalence in Japan (1.81 percent) [22]. It has been reported that many children and adolescents with developmental disorders have normal or borderline intelligence quotients [22,23]. This fact seems to be one reason that detection of developmental disorders by non-CAP professionals is delayed. The average score for the survey question about the level of difficulty in diagnosing PDD was 6.60 ± 2.1. This high score could suggest that many clinicians are concerned about the uncertainty of their PDD diagnoses as distinct from schizophrenia. The mean score on interest in schizophrenia and mood disorders in children and adolescents was 6.01 ± 2.2, measuring slightly higher than that of interest in PDD. Gaining experience in the child and adolescent psychiatric disorder cases that also commonly present among adults might attract psychiatrists to CAP and could be a factor that facilitates their entrance into CAP.

As we mentioned earlier, the subjects with developmental disorders represent a notably high proportion of new referrals to the CAP clinics. In our study, the mean scoring of interest in PDD was 5.39 ± 2.4; one quarter of the respondents answered that their interest was research-oriented. These results are consistent with the current exploratory research trends in pathophysiology and genetics [24-27]. The increased social awareness of PDD originated in 2000 when a act of violence by an adolescent with suspected PDD was widely reported by the mass media. In May 2000, a 17-year-old high-school student hijacked a bus and killed a passenger with a knife. According to the report in the newspaper, he was later diagnosed as having PDD. Similarly brutal acts by adolescents with possible PDD occurred following this and since then, the number of scientific papers on PDD has been increasing. For these reasons, PDD is a disorder that has recently attracted the most intense interest in biological psychiatry in Japan. Thus, further development of PDD studies might attract early-career psychiatrists and lead them to CAP.

Considering the results of the present study, we would like to emphasize the necessity of exposing early-career psychiatrists to more CAP cases to ensure adequate and effective recruitment into CAP. For this purpose, we recommend that all psychiatric training programs require 1) Didactics in development and psychiatric disorders in children and adolescents; 2) Provide, for example, at least two months of intensive training during residency with children and adolescents under the supervision of a psychiatrist who has been certified as an expert in child and adolescent psychiatry by the JSCAP; 3) Short-term training courses on specific topics to improve the psychiatric trainees' clinical skills to diagnose and treat child and adolescent cases. To materialize these proposals, we should think of dividing the country into several regions and provide accessible resources to all residents by establishing at least one core institute in each region. The voice of young psychiatrists should be respected to begin the discussion about a concrete action plan, and as the first step for this movement, the foundation of a section for young psychiatrists within the academic society for CAP would be helpful in order to facilitate communication with early-career psychiatrists.

We must recognize some limitations within this study. Because of a design flaw in the online questionnaire system, we were unable to calculate a precise response rate. The number of subjects surveyed was too small to draw a definitive conclusion. Considering the data provided by JMHLW which reported that the number of psychiatrists was 12,474 in 2006 (4.48 percent of all medical doctors), we estimated the number of early-career psychiatrists in Japan to be 5,063. Thus, the subjects of this study account for only 3.6 percent of all early-career psychiatrists. However, the invitation letter to this survey was sent to all 80 medical schools in Japan and the respondents were distributed throughout the country. Thus, our sample could provide a certain level of representativeness. The intention of each item on the questionnaire could be interpreted slightly differently among the respondents. Most of the respondents worked at university or general hospitals, which suggests a sampling bias. Further, survey answers were subjective assessments by the respondents, and respondents' clinical experience and diagnostic/treatment skills were not objectively evaluated by the mentors.

## Conclusion

In Japan, CAP is not a separate specialty but is instead considered a psychiatric subspecialty. Despite social demands and an urgent need to increase the number of clinicians who could address children's mental health problems, Japan continues to face a serious shortfall in its CAP workforce. In response to this situation, the Japanese government established a task force and has been providing various opportunities for general psychiatrists and paediatricians to learn about CAP and to enhance their CAP clinical skills. Our survey results demonstrated that early-career psychiatrists self-evaluated their clinical CAP experience as insufficient, and their CAP experience and CAP interest were found to correlate significantly. In order to attract more young psychiatrists to CAP, we need to provide more exposure to CAP cases during the early stages of psychiatric training. Moreover, to ensure adequate and effective CAP recruitment, we need to continue to explore those factors that can affect psychiatrists' decisions about whether to pursue a CAP career. In this respect, the present study can contribute to the further development of CAP in Japan.

## Competing interests

The authors declare that they have no competing interests.

## Authors' contributions

All authors equally contributed to the study's design and data collection, and had full access to the data. MT performed the statistical analysis and drafted the manuscript. All authors have read and approved this paper.

## References

[B1] Tsuchiya KJ, Takei N (2004). Focus on psychiatry in Japan. Br J Psychiatry.

[B2] Hayashi M, Yamazaki K (1998). Surveys on the pregraduate and postgraduate education on child and adolescent psychiatry in Japan. Psychiatry Clin Neurosci.

[B3] Crump A (2006). Suicide in Japan. Lancet.

[B4] Naito A (2007). Internet suicide in Japan: implications for child and adolescent mental health. Clin Child Psychol Psychiatry.

[B5] Honjo S, Sasaki Y, Kaneko H, Tachibana K, Murase S, Ishii T, Nishide Y, Nishide T (2003). Study on feelings of school avoidance, depression, and character tendencies among general junior high and high school students. Psychiatry Clin Neurosci.

[B6] McClure M, Shirataki S (1989). Child psychiatry in Japan. J Am Acad Child Adolesc Psychiatry.

[B7] Borovoy A (2008). Japan's hidden youths: mainstreaming the emotionally distressed in Japan. Cult Med Psychiatry.

[B8] Herrmann N, Shulman KI, Silver IL (1992). Intensive early exposure to geriatric psychiatry in residency training: impact on career choice and practice. Can J Psychiatry.

[B9] Thomas T (2008). Factors affecting career choice in psychiatry: a survey of RANZCP trainees. Australas Psychiatry.

[B10] Miyajima K, Fujisawa D, Nakagawa A, Kato T (2007). [Psychiatric training at university hospitals--a viewpoint of the first psychiatrists who were trained at the latter part of the new system]. Seishin Shinkeigaku Zasshi.

[B11] Mowbray RM, Davies BM, Biddle N (1990). Psychiatry as a career choice. Aust N Z J Psychiatry.

[B12] Shelley RK, Webb MG (1986). Does clinical clerkship alter students' attitudes to a career choice of psychiatry?. Med Educ.

[B13] Sierles FS, Yager J, Weissman SH (2003). Recruitment of U.S. medical graduates into psychiatry: reasons for optimism, sources of concern. Acad Psychiatry.

[B14] Martin VL, Bennett DS, Pitale M (2007). Medical students' interest in child psychiatry: a clerkship intervention. Acad Psychiatry.

[B15] Cutler JL, Alspector SL, Harding KJ, Wright LL, Graham MJ (2006). Medical students' perceptions of psychiatry as a career choice. Acad Psychiatry.

[B16] Likert R (1932). "A Technique for the Measurement of Attitudes". Archives of Psychology.

[B17] Brook RH, Chassin MR, Fink A, Solomon DH, Kosecoff J, Park RE (1986). A method for the detailed assessment of the appropriateness of medical technologies. Int J Technol Assess Health Care.

[B18] Keating NL, Zaslavsky AM, Ayanian JZ (1998). Physicians' experiences and beliefs regarding informal consultation. JAMA.

[B19] Allen MH, Currier GW, Hughes DH, Reyes-Harde M, Docherty JP (2001). The Expert Consensus Guideline Series. Treatment of behavioral emergencies. Postgrad Med.

[B20] Kurita H (1996). Clinical studies of pervasive developmental disorders in Japan. Psychiatry Clin Neurosci.

[B21] Kurita H (2006). Disorders of the autism spectrum. Lancet.

[B22] Kawamura Y, Takahashi O, Ishii T (2008). Reevaluating the incidence of pervasive developmental disorders: impact of elevated rates of detection through implementation of an integrated system of screening in Toyota, Japan. Psychiatry Clin Neurosci.

[B23] Teshirogi H, Tateno M, Yoneta M, Kunishige M, Maegaki Y, Takeyama Y, Tsutsumi H, Saito T (2009). WISC-III profiles of subjects with high-functioning pervasive developmental disorders who visited child and adolescent psychiatry clinics at a university hospital.

[B24] Sadakata T, Washida M, Iwayama Y, Shoji S, Sato Y, Ohkura T, Katoh-Semba R, Nakajima M, Sekine Y, Tanaka M, Nakamura K, Iwata Y, Tsuchiya KJ, Mori N, Detera-Wadleigh SD, Ichikawa H, Itohara S, Yoshikawa T, Furuichi T (2007). Autistic-like phenotypes in Cadps2-knockout mice and aberrant CADPS2 splicing in autistic patients. J Clin Invest.

[B25] Nishimura K, Nakamura K, Anitha A, Yamada K, Tsujii M, Iwayama Y, Hattori E, Toyota T, Takei N, Miyachi T, Iwata Y, Suzuki K, Matsuzaki H, Kawai M, Sekine Y, Tsuchiya K, Sugihara G, Suda S, Ouchi Y, Sugiyama T, Yoshikawa T, Mori N (2007). Genetic analyses of the brain-derived neurotrophic factor (BDNF) gene in autism. Biochem Biophys Res Commun.

[B26] Durand CM, Betancur C, Boeckers TM, Bockmann J, Chaste P, Fauchereau F, Nygren G, Rastam M, Gillberg IC, Anckarsater H, Sponheim E, Goubran-Botros H, Delorme R, Chabane N, Mouren-Simeoni MC, de Mas P, Bieth E, Roge B, Heron D, Burglen L, Gillberg C, Leboyer M, Bourgeron T (2007). Mutations in the gene encoding the synaptic scaffolding protein SHANK3 are associated with autism spectrum disorders. Nat Genet.

[B27] Nakashima N, Yamagata T, Mori M, Kuwajima M, Suwa K, Momoi MY (2009). Expression analysis and mutation detection of DLX5 and DLX6 in autism. Brain Dev.

